# Supplementary material for: The mechanical response of talin
Source: Nat Commun. 2016 Jul 7;7:11966. doi: 10.1038/ncomms11966 (PMC4941051; doi:10.1038/ncomms11966)
Supplement: Supplementary Information — Supplementary Figures 1-4 and Supplementary Table 1 [file ncomms11966-s1.pdf]

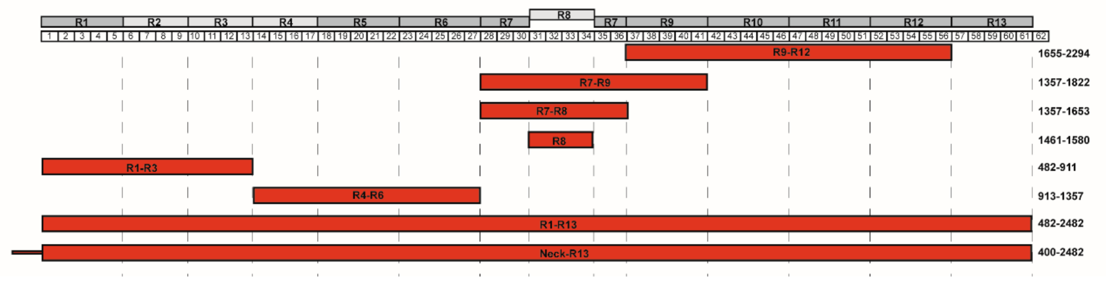

Supplementary Figure 1. Stretchable talin fragments
Schematic showing the stretchable talin rod constructs used. The mouse talin1 domain boundaries are shown.

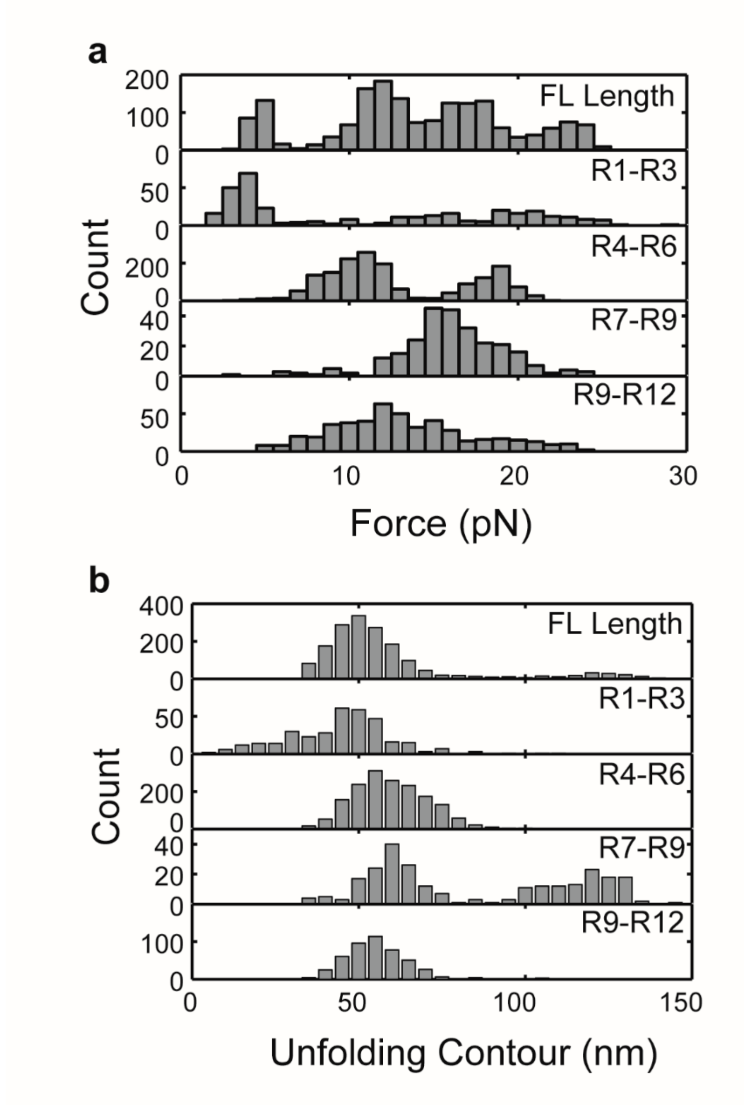

Supplementary Figure 2. Statistics of talin unfolding. (**a**) unfolding force and (**b**) contour length histogram of the talin rod domain constructs stretched in the study with 3.4-3.8 pN/s loading rates.

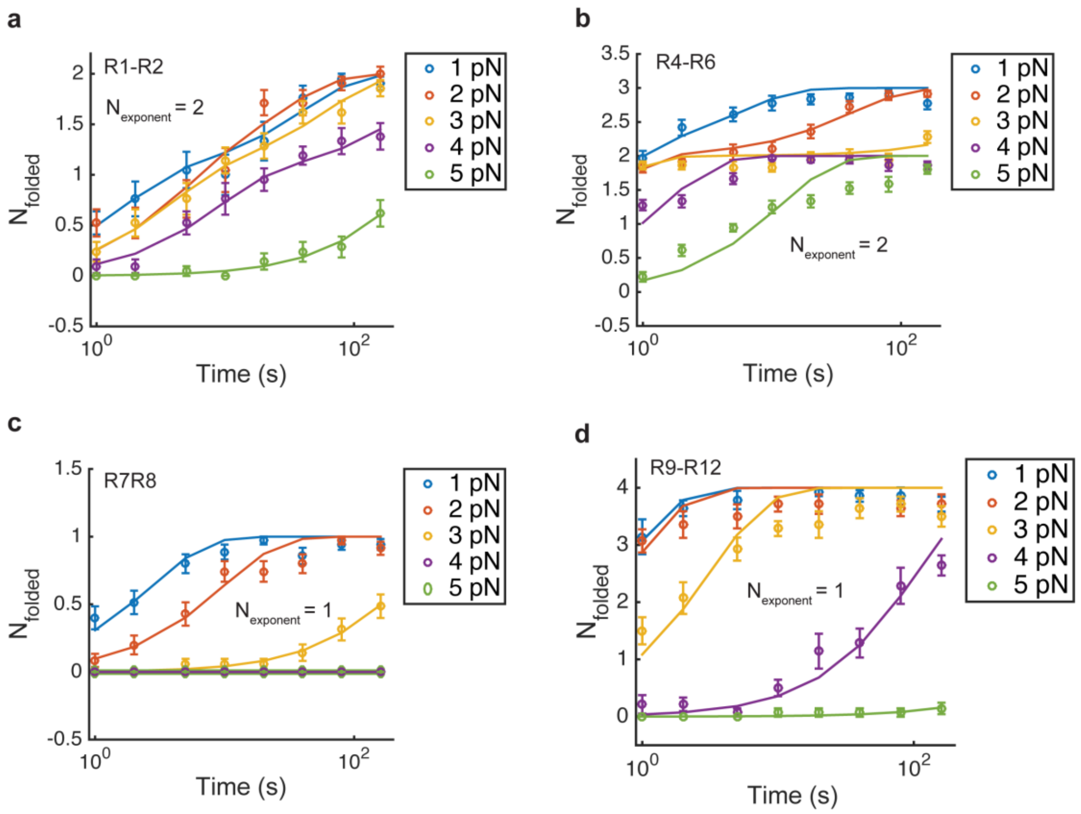

Supplementary Figure 3. The refolding kinetics of talin rod domains. The mean number of unfolding events vs. function of folding time for (**a**) R1-R2, (**b**) R4-R6, (**c**) R7-R8) and (**d**) R9-R12 constructs held at different forces. The solid lines denote exponential fitting for each force by exponential functions. The error bar denotes standard error of the mean. For R1-R2 and R4-R6 constructs, two exponents were required to fit the data.

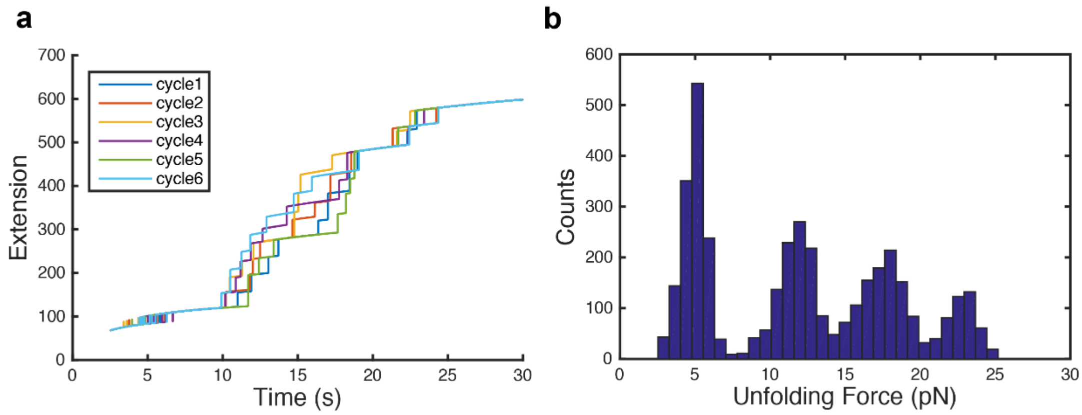

Supplementary Figure 4. Simulated FL-talin unfolding by experimental determined kinetic rates. (**a**) Simulated force-extension curves of FL talin stretched by an increasing force from 0.1 to 30 pN with a 4 pN/s constant loading rates. (**b**) The corresponding unfolding force histogram of FL-talin in (a), collected from 250 simulated traces.

Talin rod domain	1	2	3	4	5	6	7 - 8	9	10	11	12
Domain size (nm)	69.2	52.4	49.6	52.4	64	60.4	118.4	66.8	63.2	66.4	62.8
$k_{u,0}$ (s^{-1})	4.2×10^{-6}	1.7×10^{-8}	0.018	4.2×10^{-6}	2.5×10^{-5}	2.5×10^{-5}	4.2×10^{-6}	4.2×10^{-6}	2.5×10^{-5}	2.5×10^{-5}	1.7×10^{-8}
Δx_u (nm)	3.1	3.4	5.7	3.1	4.1	4.1	3.1	3.1	4.1	4.1	3.4
$k_{f,0}$ (s^{-1})	0.11	0.019	22.2	0.46	1.0	1.0	0.39	0.93	0.93	0.93	0.93
Δx_f (nm)	18.2	12.5	15.5	4.4	15.7	15.7	13.3	14.5	14.5	14.5	14.5

Supplementary Table 1. Kinetic rates of individual domains used in simulation.
Continuous grey areas denote the sequence of domains in the area that were not unambiguously assigned.
